# Challenges with using popular entertainment to address mental health: a content analysis of Netflix series *13 Reasons Why* controversy in mainstream news coverage

**DOI:** 10.3389/fpsyt.2023.1214822

**Published:** 2023-08-30

**Authors:** Hua Wang, Zhiying Yue, Divya S

**Affiliations:** ^1^Department of Communication, University at Buffalo, The State University of New York, Buffalo, NY, United States; ^2^Boston Children’s Hospital, Harvard Medical School, Boston, MA, United States; ^3^Department of Communication Studies, University of Georgia, Athens, GA, United States

**Keywords:** *13 Reasons Why*, Netflix, suicide, mental health, social impact entertainment, entertainment-education, content analysis

## Abstract

**Background:**

Mental health conditions and psychiatric disorders are among the leading causes of illness, disability, and death among young people around the globe. In the United States, teen suicide has increased by about 30% in the last decade. Raising awareness of warning signs and promoting access to mental health resources can help reduce suicide rates for at-risk youth. However, death by suicide remains a taboo topic for public discourse and societal intervention. An unconventional approach to address taboo topics in society is the use of popular media.

**Method:**

We conducted a quantitative content analysis of mainstream news reporting on the controversial Netflix series *13 Reasons Why* Season 1. Using a combination of top-down and bottom-up search strategies, our final sample consisted of 97 articles published between March 31 and May 31, 2017, from 16 media outlets in 3,150 sentences. We systematically examined the news framing in these articles in terms of content and valence, the salience of health/social issue related frames, and their compliance with the WHO guidelines.

**Results:**

Nearly a third of the content directly addressed issues of our interest: 61.6% was about suicide and 38.4% was about depression, bullying, sexual assault, and other related health/social issues; it was more negative (42.8%) than positive (17.4%). The criticism focused on the risk of suicide contagion, glamorizing teen suicide, and the portrayal of parents and educators as indifferent and incompetent. The praise was about the show raising awareness of real and difficult issues young people struggle with in their everyday life and serving as a conversation starter to spur meaningful discussions. Our evaluation of WHO guideline compliance for reporting on suicide yielded mixed results. Although we found recommended practices across all major categories, they were minimal and could be improved.

**Conclusion:**

Despite their well intentions and best efforts, the *13 Reasons Why* production team missed several critical opportunities to be better prepared and more effective in creating social impact entertainment and fostering difficult dialogs. There is an urgent need to train news reporters about established health communication guidelines and promote best practices in media reporting on sensitive topics such as suicide.

## Introduction

Mental health conditions and psychiatric disorders are among the leading causes of illness, disability, and death among young people around the globe (1). In the United States, teen suicide has increased by about 30% in the last decade, with the highest being 11.2 deaths per 100,000 15-19-year-olds in 2017–2019 (2). Although suicide is preventable, it is often associated with and complicated by factors such as social isolation, major depression, substance use, bullying, and sexual abuse, thus heightening risks for teenagers and adding billions to the economic burden of medical costs and loss of productivity every year (3–6). Raising awareness of warning signs and promoting access to mental health resources can help reduce suicide rates for at-risk youth (2). However, death by suicide remains a taboo topic for public discourse and societal intervention [e.g., (7, 8)].

An unconventional approach to address taboo topics in society is the use of popular media [e.g., (9–11)], with a rapidly growing global alliance in “social impact entertainment” in recent years (12). Creative writers and producers have been leveraging the popularity of entertainment media and the power of storytelling to engage and educate mass audiences for decades. Mental health storylines have appeared in iconic Norman Lear sitcoms such as *Maude* and *One Day at a Time* [The Norman Lear (13)], Oscar-winning movies such as *Still Alice* and *The Father* (14), and many other top rated and critically acclaimed programs on television networks and streaming platforms [e.g., (15–17)].

*13 Reasons Why* (18) is a Netflix original series adapted from Jay Asher’s young adult novel with the same title. Its Season 1 premiered on March 31, 2017, and portrayed a host of sensitive issues such as teen suicide, depression, bullying, and sexual assault (19). The story was narrated by the main character, Hannah Baker, a high school student who took her own life and left behind 13 cassette tapes explaining the 13 reasons why she died by suicide. Unlike shows that addressed suicide in popular culture before, *13 Reasons Why* released its 13-episode debut season all at once with elaborate plots filled with suspense, and twists and turns that were meant to stimulate conversations about taboo issues that people often shy away from Lawler (20) and Shef (21).

The show was quickly dubbed “the most tweeted-about show” in 2017 (22–24), went viral on other popular social media platforms (25), and got renewed by Netflix (26). In fact, it went on to complete three additional seasons. However, Season 1 also caused significant controversy and a wave of media moral panic (27, 28), with headlines like “*13 Reasons Why* tells teens they are better off dead” and “*13 Reasons Why* spreads suicide like a disease.” Scholars have looked at how this controversy set off Google Trends in suicide-related online search (29) and its potential associations with teen suicide rates in the United States (30, 31) as well as pediatric and adolescent hospital admissions and crisis helpline usage (32, 33).

News reporting by mainstream media can affect agenda setting and public opinions. A recent scoping review of television entertainment narratives in lay populations showed *13 Reasons Why* as one of the most frequently studied programs (16). Yet, there is no publication to date that has systematically investigated how the controversy was reported by mainstream news outlets when this show was first released in the United States except for one in the Canadian media (34). Our study presented here fills this research gap. Using Entman’s (35) framing theory of mass communication, we examined the three research questions: What frames did mainstream news media select to cover this controversy? How salient were the frames related to health/social issues in the mainstream news coverage of this controversy? And to what extent did mainstream news media reports comply with established guidelines? In this article, we briefly review the framing theory and its application in news media. We then provide descriptions in the method section about our sampling strategy and coding procedure before detailing the results. We conclude with a discussion about practical implications and recommendations for future endeavors.

### News framing in mass media

The concept of framing was initially developed by Goffman (36) to assist individuals in organizing and processing information in everyday life. Gitlin (37) further linked framing to mass media, particularly in the context of news coverage, where reporters employ frames to draw the public attention to specific aspects of a phenomenon. Over the past four decades, framing has been considered as a fundamental theory of mass communication and media effects (38, 39).

Entman (35) elaborated that “to frame is to select some aspects of a perceived reality and make them more salient in a communicating text, in such a way as to promote a particular problem definition, causal interpretation, moral evaluation, and/or treatment recommendation for the item described” (p. 52). Therefore, the two essential elements of news framing are *selection* and *salience*, which were the focus of the present study. The way a phenomenon is presented in the news can have significant psychological, cultural, and societal impacts [e.g., (40–45)].

Frames help the audience to locate, perceive, identify, and label the flow of information around them (36) and to narrow the available alternatives in mind (46). Several studies have emphasized the importance of news framing by focusing on the consequences of the public’s interpretation of critical events and issues [e.g., (47–49)]. For instance, Kim et al. (48) examined how newspapers covered the causes of and solutions to racial/ethnic health disparities over time. After analyzing 3,823 articles, they found that behavioral explanations, such as individual lifestyle choices and personal habits, were predominantly used to explain racial/ethnic health disparities in U.S. newspapers from 1996 to 2005. This emphasis on individual behavior overshadowed structural factors, such as systemic racism, access to healthcare, and socioeconomic conditions. The researchers concluded that this framing might create barriers and limit public solutions, as it shifts the focus away from addressing underlying systemic issues that contribute to these disparities.

### Media framing on suicide

Framing effects are also salient in mass media coverage of suicide [e.g., (50, 51)]. Mass media is often considered critical in suicide prevention (52, 53). On one hand, media coverage on suicide is often selective and biased (54). Such media stereotypes may stimulate the stigmatization of suicidal individuals and suppress people’s intention to seek professional help (55). On the other hand, copycat behavior is well-documented following media coverage of suicide, especially celebrity suicide [e.g., (56)]. Results of a meta-analysis showed a significant increase of suicide in the month following a celebrity suicide news report (53). Even the subtle language use in the news report, for example, “free death” and “self-murder” can prime problematic frame-related concepts such as greater attitudinal support for suicide (50).

### Guideline compliance

Guidelines for reporting on suicide have been developed and shared by experts and health organizations in the United States and worldwide [e.g., (57–59)]. For example, the U.S. Center for Disease Prevention and Control or CDC (57) updated its 1994 recommendations from a national workshop to suggest effective news reporting guidelines on suicide based on results of over 50 research studies. Similarly, the World Health Organization or WHO (59) released a revised edition of their 1999 booklet of resources and guidelines as the product of their decades of collaboration with the International Association for Suicide Prevention. In this 29-page booklet, WHO (59) included a one-page quick reference guide of six dos and don’ts along with examples that can easily guide content coding.

All these guidelines emphasize the importance of avoiding sensational headlines, languages that normalize or stigmatize suicide, explicit descriptions of the method or location while providing accurate information to educate the public about the facts of suicide and suicide prevention, offering specific resources to seek help, and sharing stories of how to cope with stressors in life that could lead to suicide. These recommendations of best practices are consistent across different sources. Although Carmichael and Whitley (34) chose the recommendations for Canadian journalists to adhere to best practices in the media industry, we used the WHO (59) guidelines in our study.

In summary, we were interested in how mainstream news media framed the controversy about Netflix series *13 Reasons Why* Season 1 around teen suicide and related health/social issues, and if they complied with the established guidelines for media professional. Based on the above literature review, we proposed the following research questions:

RQ1: What frames did mainstream news media select to cover this controversy?

RQ1a: What were the selected frames in terms of content?

RQ1b: What were the selected frames in terms of valence?

RQ2: How salient were the frames related to health/social issues in the mainstream news coverage of this controversy?

RQ3: To what extent did mainstream news media reports comply with the WHO guidelines?

## Materials and methods

### Sampling strategy

To create a comprehensive database of news reports, we employed a dual approach, combining both top-down and bottom-up search strategies. This allowed us to capture a diverse and inclusive range of information relevant to our research objectives. Our top-down approach focused specifically on mainstream news outlets, as they tend to be the most influential and have the broadest reach. To compile this list, we relied on circulation and popularity data (60–62) as well as expert librarian recommendations (D. Hartman, personal communication, November 2, 2017). This approach was crucial as some of these mainstream sources were not available through news aggregators such as Google News, and their inclusion was essential to thoroughly understand the media landscape surrounding our topic. From this approach, we identified a total of 18 key outlets (Table 1).

**Table 1:** Mainstream media news outlets used in this study.

Number	Title (in alphabetical order)	
1.	✓	*ABC News*
2.	✓	BBC News
3.	✓	Boston Globe
4.	✓	CBS News
5.	✓	Chicago Tribune
6.	✓	CNN
7.	✓	Daily News
8.	✓	Los Angeles Times
9.	✓	NBC News
10.	✓	New York Post
11.	✓	Newsday
12.	✓	NPR
13.		PBS
14.	✓	The New York Times
15.	✓	The New Yorker
16.		The Wall Street Journal
17.	✓	The Washington Post
18.	✓	USA Today

The news outlets with a tick mark are the ones included in our final sample.

Meanwhile, the bottom-up approach involved utilizing a custom Python script to mine all pertinent news reports from Google News. This method was employed to ensure that we gathered relevant information from a wide array of media sources, including those that may not be as well-known or easily accessible. This approach aimed to avoid potential bias by accounting for various perspectives and opinions from different media outlets. Furthermore, the bottom-up approach was designed to confirm the thoroughness and comprehensiveness of our top-down approach, ensuring that we captured an accurate representation of the mainstream media landscape and its coverage of our topic.

**Figure 1:**
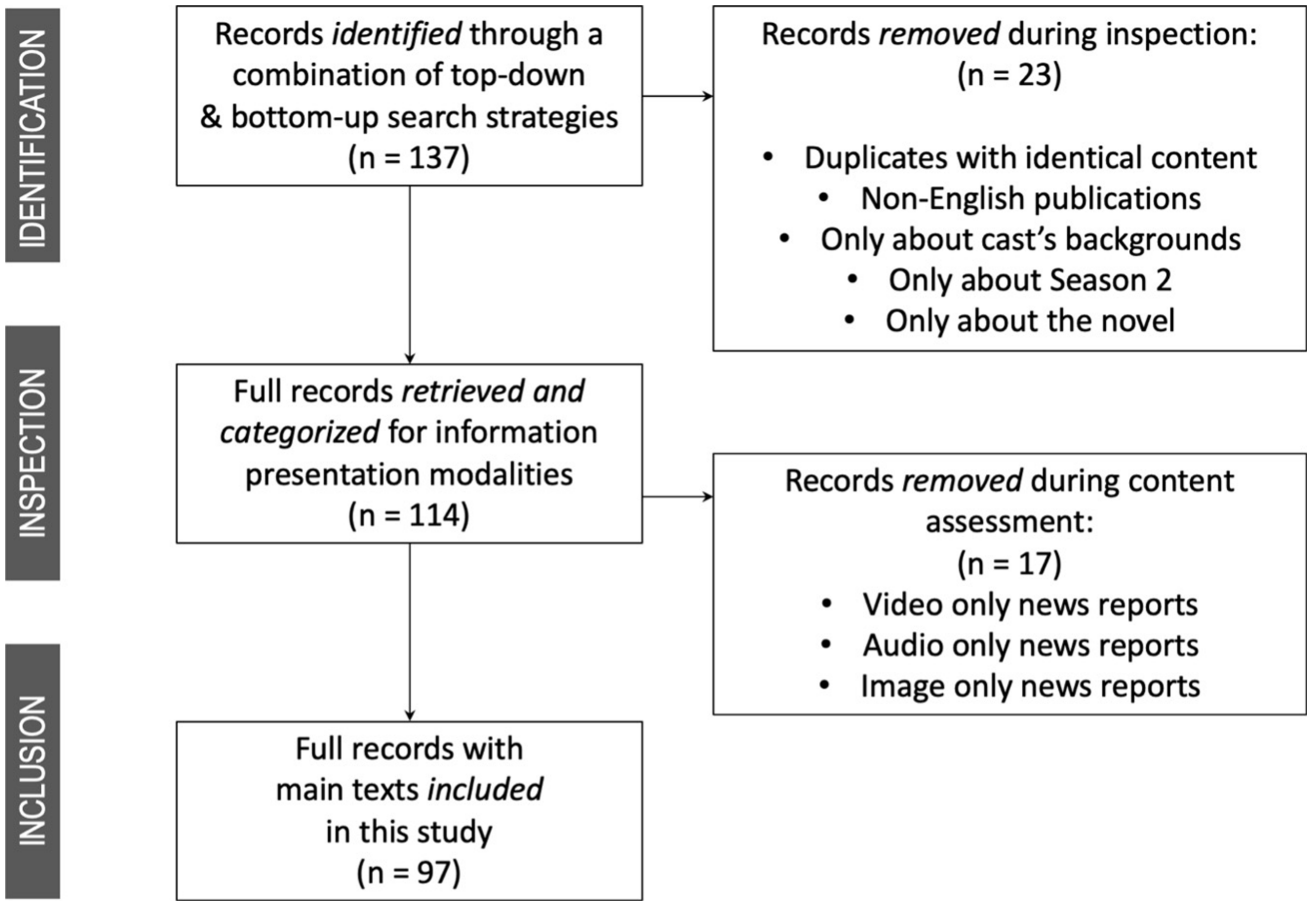
Flowchart of news report selection.

Our search criteria included a combination of keywords “*13 Reasons Why*” AND “Netflix” in the main text and a publication date between March 31 and May 31, 2017. We selected an 8-week timeframe for data retrieval, as most news articles about a particular event are typically published within the immediate days and weeks following the event due to the rapid 24/7 news cycle (63, 64). Our general search strategies, conducted between December 2017 and April 2018, initially yielded 137 news reports from 18 unique media outlets (Table 1; Figure 1). Full texts of these news reports were retrieved, some with paid subscriptions to gain access. Upon further inspection, we excluded duplicates, non-English publications, and articles that only discussed the cast’s personal backgrounds, the novel, or Season 2 without any mention of Season 1 content. This refined selection process allowed us to concentrate solely on the news coverage of controversy around Season 1 (Figure 1). Of the remaining 114 news reports, 15 were video only, one was audio only, one was image only, three were text only, and 94 were multimedia, using a combination of text and audio/image/video. Our final analytical sample consisted of 97 news reports (Figure 1; Appendix A in Supplementary material) from 16 mainstream news outlets (Table 1), which included text-based news coverage of the Netflix series *13 Reasons Why* Season 1 with a total word count of 68,601 (*min* = 115, *max* = 2,827, *Mean* = 722.12, *Median* = 640, *SD* = 486.91).

### Coding procedure

To address the research questions, we employed a quantitative content analysis (65) to examine the news reports, with individual sentences serving as our units of analysis. This approach allowed for a more precise assessment of the content by focusing on each sentence’s relevance to the research questions. We opted for human coding rather than computational methods to ensure accurate interpretations of the context, nuances, and valence of the sentences (66).

Each news report was assigned a unique document ID, with every paragraph and sentence within a paragraph in the main text marked for coding. Sentences were dummy coded based on their direct relevance to 13 Reasons Why Season 1 on Netflix and whether they pertained to the program in general or the health/social issues addressed. For issue-relevant sentences, the content was dummy coded as either a general comment or specific to a particular issue (e.g., suicide, bullying, depression, etc.). The coding criteria were as follows:

1. Relevance to Health/Social Issues: First, we evaluated if a given sentence was relevant to any specific health or social issue. (yes/no)
2. Specific Issue Identification: If a sentence was deemed relevant from the first criterion, it was then individually assessed against each of the six domains to determine its relevance. It’s crucial to note that a single sentence could be coded multiple times to see if it was relevant to each of the domain: (1) suicide, (2) depression, (3) bullying, (4) sexual assault, (5) drug use, or (6) other? (yes/no)
3. Valence of the sentence: For sentences that were relevant based on the above criteria, we then assessed their valence. This categorization was based on the nature of the content:

Positive = praise of the show;
Negative = criticism of the show;
Neutral = a plain description or statement.

4. If the sentence is relevant to suicide, is it compliant with the WHO guidelines? (yes/no)

1. Do provide resources for seeking help;
2. Do educate the public with accurate information about suicide and suicide prevention;
3. Do share stories of how to cope with stressors in life that can lead to suicide;
4. Do not use languages that sensationalize, normalizes, or stigmatizes suicide;
5. Do not explicitly describe the method used; and
6. Do not provide details about the site/location.

In our study, we also coded separately for the mention of 13ReasonsWhy.info that the show created to provide comprehensive and creditable resources for their audiences and the mention of the 29-min documentary-style video *Beyond the Reasons* that Netflix released at the same time as Season 1 where the writers, producers, cast, and expert consultants discussed how they worked together when making creative decisions about the difficult scenes related to suicide and associated health/social issues.

These coding schemes were input into an Excel file for storage and analysis. For each question, frequency counts and proportions were calculated. Two coders were trained and tested a small sample together before undertaking the coding task independently. They each analyzed the entire sample of 97 news reports. Intercoder reliability was assessed by comparing the two coders’ categorization of each unit to calculate a numerical index of their agreement (67). The average agreement percentage was 97.5%. We then calculated Krippendorff’s α as the standard reliability measure because it could be used regardless of the number of coders, levels of measurement, sample size, or presence/absence of missing data (68). The Krippendorff’s α of this study was 0.87, indicating a satisfactory degree of intercoder reliability.

### Results

To address RQ1a and RQ2, we analyzed a total of 3,150 sentences. A small portion (443/3,150; 14.1%) was found to be irrelevant to either the show or the health/social issues. More than half (1,784/3,150; 56.6%) of the sentences were directly related to *13 Reasons Why* Season 1, focusing on the show’s content, storyline, or general comments about the series. On the other hand, nearly a third (1,030/3,150; 32.8%) of the sentences were specifically related to health/social issues, such as mental health. It is important to notice that some sentences addressed both the health/social issues portrayed in the show and the show itself. As a result, these sentences were coded multiple times, leading to a total count greater than 3,150 when adding up the numbers for each category. To answer RQ1b, our analysis revealed both criticisms and praises about how the show addressed sensitive health/social issues; however, the comments were overwhelmingly more negative (441/1,030, 42.8%) than positive (179/1,030; 17.4%).

### News reporting on the portrayal of teen suicide

#### Criticism

Suicide received significant news coverage in these news reports, as expected (634/3,150; 20.1% of all sentences or 634/1,030; 61.6% of issue-related sentences). Among these sentences, over half (350/634, 55.2%) were negative and they spread across 73 articles (73/97; 75.3%) we analyzed, meaning, on average, 4.79 negative comments per article. They also accounted for a majority (350/441, 79.4%) of all negative comments in this study, including opinions and petitions advocating for the show to be completely removed from Netflix [e.g., (69, 70)].

The most frequently appeared theme was the risk of suicide contagion. Over 40 articles quoted at least one mental health expert to highlight the show’s potentially damaging impact on young audiences. For example, a Washington Post report stated that the series “sparked warnings from mental health counselors and others expressing concern about copycat behavior” (71). It was considered “irresponsible” and “could lead to copycat attempts in real life” (72). Specifically, as USA Today (73) pointed out that “the main concern is that the show arguably sensationalizes teen suicide” and “the contagion effect has been well documented as a real phenomenon for teen suicide.” Therefore, “educators, parents and school counselors are raising the alarm that the show…could even lead to copycat deaths” said in an NPR report (74). Specific examples from both youths and parents were also included. One article from Daily News described a special case about “Jackson, who admitted to attempting suicide in the past, called the show ‘extremely triggering’”; she wrote on her Instagram that “please only watch this show with caution and keep in mind that it may put you in a dark place. And if you are struggling, please do not watch it” (75). A mother in Florida even blamed “teacher’s recommendation [of] students watch *13 Reasons Why*” for her son’s “attempting to take his life” (76).

Another major criticism was that the show potentially glamorized suicide. By showing the teenagers that “taking your life is an option for exacting revenge” (77), sentences in 21 articles explicitly claimed that *13 Reasons Why* glamorized risky behaviors such as suicide [e.g., (78)]. Some mental health experts also supported this argument. For example, an NBC report quoted Dr. Tami Benton, Associate Professor at the University of Pennsylvania, “*13 Reasons Why* glamorizes suicide and makes it seem as if there is not any other option, and that is really a problem” (79). In fact, the criticism about Hannah Baker’s graphic suicide scene was so harsh and persistent that Netflix eventually gave in and removed it completely from Season 1 (80) while the producers’ moral obligations and social responsibilities were called into question (28).

Furthermore, *13 Reasons Why* was criticized for how they portrayed adult characters in the show and how they appeared “clueless,” “incompetent,” and “do not care” [e.g., (81)]. For example, the very last reason for Hannah Baker’s death by suicide as portrayed in this show was that “hours before her death, Hannah goes to see the school counselor, hints that a senior assaulted her, and cryptically says, her eyes welling with tears, that “I need everything to stop, people, life.” He hands her tissues but does not conduct a suicide-risk assessment or consider hospitalization.” The National Association of School Psychologists released their advisory for educators regarding this controversial entertainment program (81).

#### Praise/acknowledgement

Only 62 sentences (62/3,150; 2.0% of all sentences and 62/1,030; 6.0% of issue-related sentence) in 28 articles (28/97; 28.9%) were positive. Some acknowledged the efforts that the screenwriters and producers made to carefully portray suicide. In particular, the show’s creator Brian Yorkey was quoted that “We worked very hard not to be gratuitous, but we did want it to be painful to watch because we wanted it to be very clear that there is nothing, in any way, worthwhile about suicide” (82). In addition, *13 Reasons Why*, rated TV-MA, was praised for raising awareness of “the problem teenagers can face” (83) by “viewers and critics alike” (84). For example, Ellen DeGeneres, a big fan of the show, was quoted, “I think it’s amazing. I think the reason it’s resonating is most people – and not just teenagers – but most people watching it – no matter how old you are – has experienced some type of that abuse” (85). A school counselor with personal connections to the issue pointed out that, “Teenage suicide is real and needs to be discussed. The fact that this series created engagement around the topic is positive. I hope that parents, schools, and students use it to start a conversation” (86).

Indeed, some educators did leverage this opportunity to host events to talk about how they were relating to the issues portrayed in the show; and at some schools that had been dealing with teen suicides, the educator was quoted “I would never endorse banning students from watching the show. We have been working very hard on trying to reduce the stigma of any kind of adolescent mental health issue. If we in any way say that the show is not OK to talk about, that might inadvertently be sending a message that it’s not OK to talk about feeling sad or suicide [e.g., (69)]. Only 13 sentences (13/3,150; 0.4% of all sentences and 13/1,030; 1.3% of issue-related sentences) in 13 articles (13/97; 13.4%) mentioned *Beyond the Reasons*, a 29-min documentary-style video, that accompanied the release of Season 1 where the production team and expert consultants discussed how they worked together to inform the final creative decisions.

### News reporting on the portrayal of other suicide-related health/social issues

A total of 396 sentences (396/3,150; 12.6% of all sentences and 396/1,030; 38.4% of issue-related sentences) were about other social/health issues related to teen suicide portrayed in the show: depression, bullying, and sexual assault [e.g., (87)]. A total of 91 sentences were negative and raised concerns. For example, some criticized that the show characterized “parents and educators as ineffectual at best” and failing to “mention the primary – and treatable cause of suicide – depression” (88). Others reported the criticism regarding “its explicit content addressing topics like rape and bullying” (75).

In total, 117 sentences across 10 articles were positive. Generally, the show spoke to “the feeling of isolation, of hopelessness, of frustration and anger that are familiar to so many teenagers” and it addressed “rape, drugs and bullying” while being “real,” “frank and honest and will start needed dialog. It opens the door to a conversation” (70). A school psychologist and CEO of Real Psychology, Dr. Susan Lipkins noted that “many contemporary programs, including *13 Reasons Why*, are highlighting awareness about emotional problems and psychological issues, which helps stimulate conversations about these topics” (84). In fact, it did prompt parents to talk to their teenage children about some of these difficult issues they experience in life [e.g., (25)].

Notably, among the 117 positive comments, a large portion (87 sentences, 74.4%) pertained to parent–child communication. For instance, a News Daily report (89) highlighted “the efforts of the Manhattan-based Jed Foundation, which aims to prevent teen suicide, and has developed a tipsheet of talking points related to the show “13 Reasons Why”. This resource is designed to foster open dialog between parents and their children, facilitating discussions on the story’s themes and implications. Another example was a CBS news report (90) which emphasized the show’s intended purpose as a catalyst for realistic family conversations. One parent of a 13-year-old girl, Knapp, expressed appreciation for the difficult discussion that the show prompted, but also wished they had been privy to these conversations earlier. The prevalence of such sentiments in our analysis demonstrated the perceived value of addressing challenging topics through media and fostering constructive communication within families.

### Compliance with who guidelines

To answer RQ3, we calculated the sentences and articles that complied with the dos and don’ts from the WHO guidelines that applied to this study. We found 56 sentences (56/3,150; 1.8%. of all sentences and 56/1,030; 5.4% of issue-related sentences) in 32 articles (32/97; 33.0%) providing resources for seeking help. And of those, only 15 statements in 15 articles acknowledged the 13ReasonsWhy.info website where comprehensive resources were provided for suicide prevention and other associated mental health concerns and social issues. There were 63 sentences (63/3,150; 2.0% of all sentences and 63/1,030; 6.1% of issue-related sentences) in 26 articles (26/97; 26.8%) that stated facts and research findings to educate the public about suicide and suicide prevention, citing reports from CDC and scientific evidence from social and psychological studies. A total of 14 stories were shared across 12 articles (12/97; 12.4%) about how people took measures to cope with life stressors and risk factors while being sensitive and mindful about working on the issue of suicide in the media.

On the other hand, we found 13 titles of the news reports (13/97; 13.4%) to demonstrate a sensational tendency. In addition, 27 sentences (27/1,030; 2.6% of issue-related sentences) in 25 articles (25/97; 25.8%) used stigmatizing expressions such as “committed suicide” whereas 21 sentences (21/1,030; 2.0% of issue-related sentences) in 19 articles (19/97; 19.6%) used phrases such as “died/death by suicide” or “took his/her life” as recommended in the guidelines, including five articles that had mixed practices. Furthermore, 19 sentences (19/1,030; 1.8% of issue-related sentences) in 15 articles (15/97; 15.5%) explicitly described the suicide method and nine sentences (9/1,030; 0.9% of issue-related sentences) in eight articles (8/97; 8.2%) detailed the suicide site/location used by Hannah Baker in *13 Reasons Why* or in real life suicide cases. Among all 97 articles, only three (3/97; 3.1%) acknowledged the existence of guidelines for reporting on suicide by media professionals and only four articles (4/97; 4.1%) included a warning at the start of their news stories about including show spoilers and potentially triggering descriptions. Table 2 shows sample practices of these dos and don’ts.

**Table 2:** Sample practices of news reporting on controversial Netflix series *13 Reasons Why* for WHO guideline compliance.

DOs	DON’Ts
DO provide resources about seeking help	Do not use languages that sensitize or stigmatize suicide
✓ What I’ve learnt from @13ReasonsWhy is that if you know anyone who is suicidal or cutting themselves, please speak up and get them help. If you are thinking about suicide, you can call the National Suicide Prevention Lifeline 24/7 at 1–800–273-8,255. You can also text the Crisis Text Line at 741–741 to connect with a trained crisis counselor right away. ✓ The production team said they consulted with mental health professionals extensively while making the series and provide suicide prevention resources and information on crisis hotlines in more than 35 countries on the website 13ReasonsWhy.info.	✗ ‘13 Reasons Why’ tells teens they are better off dead ✗ Especially the scenes where they.. the suicide scene. It’s almost like, a how-to for committing suicide. ✓ Parents around the country are weighing in on the controversial new Netflix series about a high school student who dies by suicide. ✓ The 13-part series, based on a 2007 young adult novel of the same name, revolves around the story of 17-year-old Hannah Baker, who takes her own life and leaves behind audio recordings. The series deals with bullying, depression, sexual assault and suicide.

DO educate the public about the facts of suicide and suicide prevention, without spreading the myths	Do not explicitly describe the method used for suicide
✓ According to the CDC, the rate of suicide deaths among children between the ages of 10 and 14 has doubled since 2007. ✓ Suicide is the second leading cause of death among those ages 15 to 34, according to the U.S. Centers for Disease Control and Prevention.	✗ The Netflix series, which may be renewed for a second season, is based on the 2007 young adult novel “Thirteen Reasons Why,” by Jay Asher. It includes a graphic scene in which Hannah kills herself with a razor. ✗ He had hanged himself in the middle of the night with his blue Tae Kwon Do belt. He was 20 years old.

DO report stories of how to cope with life stressors or suicidal thoughts, and how to get help	Do not provide details about the site or location of suicide
✓ Oxford senior Kayla Manzella is one of the 13 students who recorded stories for the project. Manzella talked about being bullied her freshman year after joining the JV volleyball team. This is part of the recording that played for all 1,800 students during the morning announcements. ✓ Precautions were also made to ensure the cast had adequate resources during the grueling production. Therapists who work with suicide survivors were on hand to answer questions. Therapy dogs made a rotation on set.	✗ Toward the end of “13 Reasons Why,” the main character’s death is shown, as she sits in the tub before taking her life. Her parents later find her. ✗ The high schoolers’ project, which they have aptly named “13 Reasons Why Not,” is also in memory of Megan Abbott, a freshman who committed suicide in 2013 and was found in a wooded area behind the school.

## Discussion

### Major findings

We set out to conduct a quantitative content analysis and systematically examine how mainstream news media reported on the controversial Netflix series *13 Reasons Why* within the first two months of its Season 1 debut. Our final sample consisted of 97 articles in 3,150 sentences, of which nearly a third (1,030 sentences) were directly addressing the taboo and sensitive issues of our interest. Within this most relevant content we analyzed, 61.6% was about suicide and 38.4% was about depression, bullying, sexual assault, and other related health/social issues; 42.8% was negative, 39.8% was neutral, and 17.4% was positive. Many comments criticized the show for the risk of suicide contagion, glamorizing teen suicide, and their portrayal of parents and educators as indifferent and incompetent. Fewer comments praised the show for raising awareness of real and difficult issues young people struggle with in their everyday life and for serving as a conversation starter to spur public discussions that people typically shy away from and stimulating meaning parent–child and teacher-student dialogs. Only 13 articles acknowledged the *Beyond the Reasons* video that Netflix released at the same time to accompany Season 1 and provide insights from the creative professionals and their expert consultants.

Our evaluation of these news articles’ compliance with the WHO guidelines for reporting on suicide yielded mixed results. Although we found recommended practices across all major categories in our sample, a rather small fraction of the content (5.4% of all issue-related sentences) and one in three articles included mental health resources, and only 15 of them mentioned the 13ReasonsWhy.info website that the show promoted along with Season 1. Similarly, limited educational content (6.1% of issue-related sentence) and less than one in five articles included facts to help the public gain knowledge about suicide. And hopeful stories and positive examples of coping strategies were rare. Consistent with the findings from the content analysis of Canadian media reports (34), most of the mainstream news reporting in the United States also followed the guidelines to avoid stigmatizing languages and explicit descriptions of suicide method or location. However, there was still a decent number of articles that violated the guidelines. Perhaps what was even more concerning was that only three articles had a brief mention of any such guidelines and only four articles began with a warning about the sensitive nature of the topic.

### Practical implications for writers and producers

For creative media professionals interested in developing social impact entertainment, our findings suggested that the mainstream media coverage skewed negatively toward the public health concerns for young audiences especially at-risk and vulnerable youths, despite the writers and producers’ well intentions and best efforts. The series creator Brian Yorkey was quoted in an interview that, “I always believe talking about things is better than silence” (69). While that was true and *13 Reasons Why* did take both social media and mainstream media by storm and got everyone talking, the creative team missed several critical opportunities to be better prepared and more effective in fostering such difficult dialogs.

First, Netflix only added viewer warning cards after several weeks of criticism (81). The show was rated TV-MA for mature audiences due to depictions of depression, sexual assault, and suicide. Its content might be thought-provoking for healthy young adults but emotionally and psychologically challenging for those who are already struggling and vulnerable (91). One death or one crisis is still one too many. Given the sensitive nature of the topics portrayed, the intensity of binge-watching and its triggering potential, and the lack of viewer access regulation on streaming platforms, proper warnings are necessary to appear at the start of each episode for viewer discretion advisory.

Second, although the production team did create a comprehensive resource website 13ReasonsWhy.info and include a few cut scenes about recognizing early signs of suicidal ideation and mental health problems (19), their recognition was minimal in the mainstream news coverage. More content about mental health resources and helplines for suicide prevention can be incorporated into the narratives of the show and be placed more prominently to truly break the silence and promote help seeking behaviors among vulnerable youths.

Third, several experts pointed out that the problem they had with the show’s suicide storytelling approach was its lack of a viable alternative, misleading young audiences to believe that adults do not care, peers are cruel, and the only solution to their struggles was to take their own lives (75). Also, as Kaufman (69) reported, “A lot of kids who had seen it were really focusing on the positive message — “I realize that even the little things I do can affect people and I think I’m more conscious of my behavior now.” But when I would follow up, asking them what they could do as a positive action, a lot of them said they were not sure and wish they knew.” Decades of evidence-based practices in entertainment-education worldwide have demonstrated the power of positive behavioral modeling through relatable characters and emotionally engrossing plots that can motivate and inspire the audiences to change their attitudes, beliefs, and actions (9–11). Transmedia edutainment is an innovative strategy for narrative engagement across different planforms (92, 93). Additional and complementary narrative components through social media or other digital apps could have been used to invite and encourage viewers to come up with alternative plots, effective coping strategies, and non-linear storytelling suggestions.

### Practical implications for news reporters

For journalists working in the domains of public health and popular entertainment, our findings revealed important gaps in knowledge and practice. There was barely any mention of guidelines or inclusion of warnings in the media coverage of *13 Reasons Why* controversy in mainstream news outlets that people respect and trust. And a mix of desirable and undesirable practices as shown in the languages used in the news articles suggest that the writers were not conscious about the unintended connotations or impact of their word choices. There is an urgent need for professional training to bring established health communication guidelines to their attention and promote best practices in media reporting on sensitive topics such as suicide.

In addition, some authors admitted not having watched the show, or at least not in its entirety, before writing their article. The rapid news cycles these days with considerable competition in the “attention economy” can easily put high pressure on journalists to produce catchy news stories without having enough time or support to do their “homework.” We certainly noticed errors and inconsistencies in writing during our data analysis. It was also hard to identify original reporting when identical or overlapping materials frequently appeared around the same time to cover the same phenomenon and not everyone would cite their sources. News reporters can benefit from friendly reminders about ethical codes of conduct to avoid falling into the traps of yellow journalism.

### Practical implications for media researchers

Our content analysis of news coverage on *13 Reasons Why* offers a critical examination of how media outlets frame and discuss the show’s portrayal of the sensitive issue of teen suicide. By analyzing a diverse range of news articles, we provide valuable insights into the media’s commentary on this complex topic and its impact on the public’s perception of the show. Importantly, our study sheds light on the ways in which media framing may influence not only how people view the show itself but also the potential effects of the show on public understanding of mental health and suicide. In this context, our study serves as a reminder that when examining media effects, it is crucial to consider exogenous factors such as news coverage and media framing, as they may shape audience perceptions and induce dialog or public discussion on sensitive issues. By taking these factors into account, we contribute to a more comprehensive understanding of media effects and the broader discourse on mental health awareness and responsible media portrayal.

One notable finding from our analysis is the significant portion of positive comments that emphasize the potential of 13 Reasons Why to open conversations, especially between adolescents and parents. The show, while controversial, has undeniably sparked discussions around mental health and suicide. This presents an opportunity for further development of entertainment-education programs that can effectively address sensitive topics and facilitate meaningful conversations among parents, educators, and young people. Parents and educators should seize this opportunity to engage in an open and honest discussion about the situations depicted in the show, correct misconceptions, and provide comfort and support to adolescents who may experience negative emotions. By doing so, they can harness the attention generated by the show to promote mental health awareness, educate young people bout the complex issues surrounding suicide, and foster a supportive environment for those who may be struggling.

It is also crucial to highlight the importance of examining news coverage when evaluating the impact of any media program or intervention. Many studies tend to focus solely on the relationship between exposure to the program and the audience’s perception, without considering exogenous factors such as mainstream news coverage. However, such coverage, can significantly shape, reinforce, or even alter people’s perceptions of a media program. By examining the news coverage and its potential influence on audience perceptions, we can gain a more comprehensive understanding of media effect and develop more effective strategies for leveraging media interventions to achieve desired outcomes in various contexts.

In addition to news coverage, future research could also consider the power of social media in shaping audience perceptions of media programs and interventions. Public discussions on social media platforms play a significant role in how people perceive and react to media content, adding another layer of complexity to understanding media effects. By examining the indirect influences of social media conversations, researchers can better understand the multifaceted nature of media effects and explore new avenues for leveraging media programs and interventions to achieve desired outcomes. This study focuses on analyzing news media coverage, but social media presents a promising direction for future exploration.

### Limitations and future research

This study is not without limitations. We acknowledge that, by some measure, there were hundreds of thousands of news reports surrounding the controversial Netflix series *13 Reasons Why* weeks and months after its Season 1 premiere [e.g., (29)]. Our sampling strategy focused on English mainstream news outlets within an 8-week period and using circulation and popularity as proxies for respectable reputation and perceived public trust, implying the potential impact on their readers’ knowledge, attitudes, and behavior. In our sample, we did not distinguish different types of publications in the news such as editorials versus op-eds or letters to the editor. Nor did we distinguish different writing styles in terms of length of sentences or essays versus Q&A. These sampling and analytical choices may have excluded news reports that could be unique, diverse, and complementary in terms of language, publication time, content structure, and voices from culturally and linguistically diverse groups. These limitations could have affected the frequency counts in our results using sentences as units of analysis. Future research may include a citation network analysis, thematic analysis, or longitudinal data analysis for additional insights into the public debate over this show’s controversy to better understand the role of news framing in mass media on public opinions and health promotion of difficult issues.

## Data availability statement

The original contributions presented in the study are included in the article/Supplementary material, further inquiries can be directed to the corresponding author.

## Author contributions

HW: study conceptualization, research design, data collection supervision, data coding and analysis, manuscript writing, generating Tables 1, 2, Figure 1, and Appendix A in Supplementary material. ZY: data collection, content coding, data analysis, and manuscript writing. DS: content coding and manuscript development. All authors contributed to the article and approved the submitted version.

## Conflict of interest

The authors declare that the research was conducted in the absence of any commercial or financial relationships that could be construed as a potential conflict of interest.

## Publisher’s note

All claims expressed in this article are solely those of the authors and do not necessarily represent those of their affiliated organizations, or those of the publisher, the editors and the reviewers. Any product that may be evaluated in this article, or claim that may be made by its manufacturer, is not guaranteed or endorsed by the publisher.
